# Spatiotemporal Dynamics of Cortical Representations during and after Stimulus Presentation

**DOI:** 10.3389/fnsys.2016.00042

**Published:** 2016-05-09

**Authors:** Marieke E. van de Nieuwenhuijzen, Eva W. P. van den Borne, Ole Jensen, Marcel A. J. van Gerven

**Affiliations:** Donders Institute for Brain, Cognition and Behaviour, Radboud UniversityNijmegen, Netherlands

**Keywords:** cortical representations, visual perception, visual working memory, classification analysis, MEG

## Abstract

Visual perception is a spatiotemporally complex process. In this study, we investigated cortical dynamics during and after stimulus presentation. We observed that visual category information related to the difference between faces and objects became apparent in the occipital lobe after 63 ms. Within the next 110 ms, activation spread out to include the temporal lobe before returning to residing mainly in the occipital lobe again. After stimulus offset, a peak in information was observed, comparable to the peak after stimulus onset. Moreover, similar processes, albeit not identical, seemed to underlie both peaks. Information about the categorical identity of the stimulus remained present until 677 ms after stimulus offset, during which period the stimulus had to be retained in working memory. Activation patterns initially resembled those observed during stimulus presentation. After about 200 ms, however, this representation changed and class-specific activity became more equally distributed over the four lobes. These results show that, although there are common processes underlying stimulus representation both during and after stimulus presentation, these representations change depending on the specific stage of perception and maintenance.

## Introduction

In daily life, we are confronted with a rapid succession of visual stimuli. Processing of this endless stream of visual input has been shown to be a fast process that commences well within 100 ms after stimulus onset and activates a host of different brain regions (e.g., Seeck et al., 1997; Bacon-Macé et al., 2005; Kirchner et al., 2009; Ramkumar et al., 2013; Van de Nieuwenhuijzen et al., 2013; Isik et al., 2014; Salti et al., 2015). During this processing, stimulus information is thought to progress through the various brain areas related to the various stages of the visual ventral information stream, including early visual areas such as occipital cortex, and higher-order inferior temporal cortices (Thorpe and Fabre-Thorpe, 2001; Kirchner and Thorpe, 2006; Serre et al., 2007), as well as areas along the dorsal stream in the case of tools, terminating in the parietal lobe (Almeida et al., 2008, 2010; Sakuraba et al., 2012).

Previous studies have shown that the spatiotemporal progression of visual category information can be extracted from magnetoencephalography (MEG) data (Van de Nieuwenhuijzen et al., 2013; Cichy et al., 2014). In this study, we aimed to map this progression in detail to determine how information about stimulus category flows through the brain in terms of space and time. We maximized spatial and temporal resolution as well as sensitivity by applying a multivariate classification technique to MEG data during visual perception of a to-be-memorized image. Classification methods are more sensitive than univariate methods for probing distributed patterns of brain activity (Lange et al., 1999; Kriegeskorte et al., 2006; Kriegeskorte, 2011). We applied this method to every single time point, obtaining a temporal resolution at the millisecond level. In the spatial domain, we extracted the activation patterns underlying successful distinction between visual categories and thus signifying information content (Haufe et al., 2014). Source reconstruction of these activation patterns provided information about the specific underlying sources at each single time point.

In addition to the spatiotemporal dynamics of visual category information during stimulus presentation, we assessed how the information flow behaved after the stimulus had disappeared from view, but still had to be maintained in memory. In some studies (Carlson et al., 2011, 2013; Ramkumar et al., 2013; Clarke et al., 2015), though not all (Liu et al., 2009; Simanova et al., 2010; Bode et al., 2012; Van Gerven et al., 2013; Cichy et al., 2014) the period after stimulus offset starts with a short time window during which information about stimulus content peaks, similar to the peak in representational content commonly observed after stimulus onset. We ask whether this offset peak can be explained in terms of a mere undershoot of neuronal activation, or whether this peak, as well as the rest of the period after stimulus offset, could play a role in the encoding of the previously shown stimulus to working memory.

## Materials and methods

### Subjects

MEG data of 30 healthy subjects (17 men; 23 right-handed; mean age: 24.50; *SD* = 8.01) were collected. Datasets of two subjects were excluded, one due to malfunctioning of the MEG system and one because an MRI scan could not be acquired, rendering source-space analyses infeasible. This resulted in data of 28 subjects (16 men; 22 right-handed; mean age: 22.93; *SD* = 2.80). All subjects had normal or corrected-to-normal vision, and gave written informed consent. The study was approved by the local ethics review board (CMO region Arnhem-Nijmegen, The Netherlands).

### Paradigm and stimuli

The data used in this study were part of an extended delayed match-to-sample task, in which subjects had to memorize images belonging to different categories (neutral faces, handheld objects, and handwritten letters). These images were presented at the center of the screen, spanning 6° of the visual field. Each category consisted of 42 different images, divided into two subcategories: male and female faces, tools and kitchen utensils, and the letters I and N. Face images were derived from the Karolinska Directed Emotional Face dataset (KDEF; images F1, F2, F3, F6, F7, F8, F9, F10, F11, F13, F17, F19, F20, F21, F22, F24, F25, F26, F27, F30, F33, M37, M39, M41, M42, M43, M44, M45, M46, M47, M52, M54, M56, M58, M59, M60, M61, M62, M63, M64, M65, M67; Lundqvist et al., 1998; Goeleven et al., 2008). Object images came from the Bank of Standardized Stimuli (BOSS; Brodeur et al., 2010). Letter images were derived from the Tilburg Character dataset (TICH; Van der Maaten, 2009). All images were resized and cropped to 300 × 300 pixels, converted to grayscale, and corrected for luminance with the SHINE toolbox for MATLAB (Willenbockel et al., 2010).

**Figure 1:**
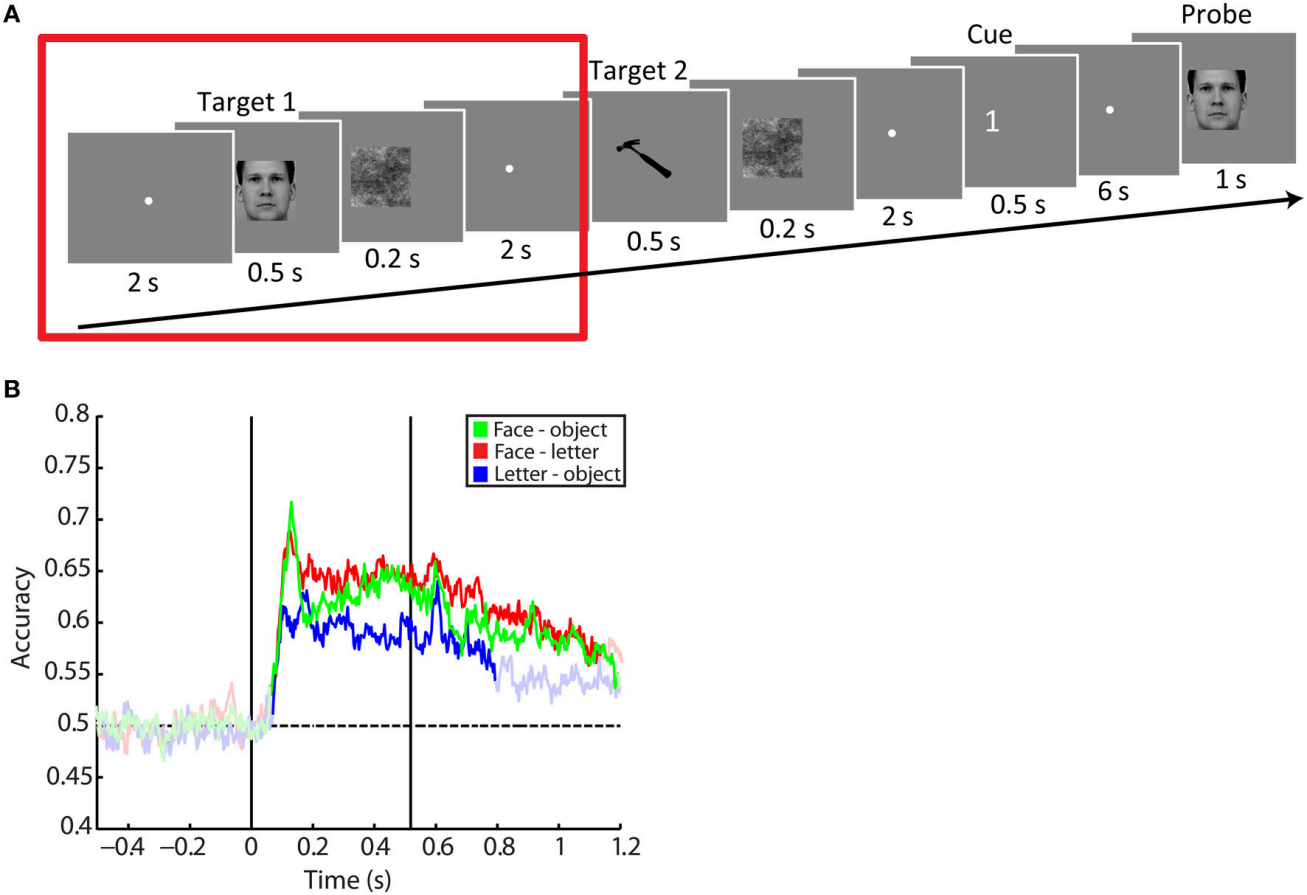
**Task design and accuracies over time. (A)** Task design. Subjects were shown two images of different classes (faces, objects or letters) per trial. These images were followed by a delay period. After the second delay period a cue was presented, informing the subject which target stimulus had to be kept in memory. After a final delay period, the subject was probed with a same-difference task. In this study we only used data of the period around the presentation of the first target, depicted as the period enclosed by the red square. All presentation timings were in reality 16.7 ms longer than depicted due to a fixed delay in stimulus presentation. The face image belongs to the KDEF dataset (M64; Lundqvist et al., 1998), the tool image is part of the BOSS set (Brodeur et al., 2010; CC BY-SA 3.0). **(B)** Averaged accuracy traces for the face-letter (green), face-object (red), and letter-object (blue) contrasts. The first black vertical line indicates stimulus onset, the second line coincides with stimulus offset. Bright colors indicate accuracies significantly above the accuracies obtained at baseline (before stimulus onset). We only tested post-stimulus onset accuracies against baseline. The dashed vertical line denotes the chance-level value of 0.5.

A schematic of the task is shown in Figure 1A. Each trial began with an inter-trial interval of 2.017 s, during which a fixation dot with a diameter of 0.5° was presented at the center of the screen. Next, a target image was presented for 0.517 s. This was followed in the next 0.217 s by a noise mask of the same size as the image, in an attempt to decrease the bleeding of the visual image into the following delay period. The delay period itself lasted 2.017 s. During this delay period only the fixation dot was presented. In this study, we focus on the perception of this first target. The additional 16.7 ms in stimulus timing were due to a fixed delay in stimulus presentation related to the 60 Hz refresh rate of the projector. Trials in which the timing of the stimulus presentation turned out to be too inaccurate, i.e., in which the actual trial length deviated more than 0.8 ms from the desired trial length, were discarded.

In the remainder of the task, which was not included in the analyses of this study, another target image, belonging to a different category than the first one, was presented, and again followed by a mask and a delay period. Subjects were then presented with a retro-cue, indicating either 1, 2, or 1+2, meaning that from that point onwards only the first, only the second, or both targets had to be maintained in memory. After a final delay period of 6 s, subjects were probed with an image that was either the same as the target that had to be memorized, or a different image from the same subcategory. When both targets had to be maintained, either the first or second target was probed. Subjects had to indicate whether the probe was the same or different from the target image. Responses were made with the dominant hand by pressing the left (same) or right (different) button on a button box. Subjects had 1 s to respond. Feedback was given after each trial.

Trials were presented in 18 blocks of 14 trials. Each block ended with a subject-paced break during which the progress of the task as well as the average reaction time and percentage correct thus far were displayed. Twenty seconds of resting state, during which subjects were solely required to look at the fixation dot, preceded blocks 1, 7, and 13. These data were not analyzed in this study. All images were presented twice as the first target, and twice as the second target. The order of the targets was pseudo-randomized, such that all combinations of Target 1 and Target 2 categories occurred equally often. Before the start of the actual task, subjects were presented with eight practice trials with different target stimuli than those that were used in the actual experiment.

Stimuli were presented with Presentation software (Version 16.2, Neurobehavioral Systems, Inc.) via an LCD projector with a refresh rate of 60 Hz outside the magnetically shielded room, and projected on a translucent screen via two front-silvered mirrors. The projector lag was measured at 35 ms, which was corrected for by shifting the time axis of the data accordingly.

### MEG recordings

MEG data were recorded with a 275-sensor whole-head system (CTF Systems Inc., Port Coquitlam, Canada) at a sampling rate of 1200 Hz. Data of two sensors (MRF66 and MRO52) were not recorded due to sensor malfunctioning. Subjects were seated in a dark, magnetically shielded room. Head location was measured with two coils in the ears and one on the nasion. To reduce head motion, cushions were fitted between the head and the helmet, and a neck brace was used to stabilize the head. Head motion was monitored online throughout the task with a real-time head localizer (Stolk et al., 2013). If subjects had moved their head more than 5 mm from the starting position they were repositioned during block breaks.

A continuous electrooculogram (EOG) was recorded with four electrodes around the eyes: two above and below the left eye for vertical EOG, as well as one left of the left eye and one right of the right eye for horizontal EOG. Furthermore, an electrocardiogram was recorded with an electrode on the left collarbone and one below the right ribs. The ground electrode was placed at the left mastoid. Eye motion was additionally measured with an Eye Link SR Research Eye Tracker.

### Preprocessing

Data were analyzed with MATLAB version R2013a and the open source MATLAB toolbox FieldTrip for analysis of neuroimaging data (Oostenveld et al., 2011), as well as FreeSurfer (Version 5.3.0; Fischl, 2012) and MNE-Suite (Version 2.7.0; http://www.martinos.org/mne/stable/index.html) for some steps of source localization (see below).

Data were low-pass filtered at 100 Hz and line noise was removed with a 50 Hz notch filter. Environmental noise, measured with third-order synthetic gradiometers, was subtracted from the data.

Only trials in which a correct response to the probe was given were included, as an incorrect behavioral response could indicate a failure in perception of the target stimulus. The data were then visually inspected, and trials containing artifacts caused by muscle activity and SQUID jumps were rejected. This resulted in the inclusion of on average 70.50 (*SD* = 9.64) trials in which faces were presented, 70.93 (*SD* = 8.36) trials in which letters were presented, and 69.71 (*SD* = 8.45) trials in which objects were presented. Faulty sensors were removed based on visual identification.

Next, data were downsampled to 300 Hz, a baseline correction was performed per trial on the period of 800–600 ms before stimulus onset, and independent component analysis (ICA) was performed. Eye motion and heart beat components were identified visually and removed from the data. The decomposition was then backprojected to sensor-level data. As this study focused on the presentation of the first target only, trials were henceforth defined as data between 500 ms before the onset of the first target and 1200 ms thereafter. The resulting time-domain data were used for further analyses.

### Classification analysis

Classification analyses were performed with an elastic net logistic regression algorithm (Friedman et al., 2010). This algorithm maximizes the log-likelihood, taking into account the elastic net penalty:

$$\begin{array}{l} P_{\alpha }\left(b\right)=\;\overset{p}{\underset{j=1}{\sum }}\left(\frac{1}{2}\left(1-\alpha \right)b_{j}^{2}+\;\alpha |b_{j}|\right) \end{array}$$

where *p* is the number of sensors and *b* is the vector of regression coefficients. In this penalty term, the mixing parameter α combines L_1_ and L_2_ regularization such that α = 1 leads to L_1_-regularized logistic regression, and α = 0 results in L_2_-regularized regression. In this study α was set to 0.01. The influence of this penalty on the coefficient estimates was controlled by a parameter λ, which was optimized using a nested cross-validation procedure. Data were standardized to have zero mean and unit standard deviation before classification, apart from transfer learning (see below), where this was done separately for each fold.

Binary classifications were performed between faces and letters, faces and objects, and letters and objects. Classification accuracy was defined as the proportion of correctly classified trials. Classifier performance was validated using five-fold cross-validation. This ensures that the classifier was always tested on trials it was not trained on, thus preventing double dipping (Kriegeskorte et al., 2009).

This classification paradigm was applied for each individual subject to every time point of the sensor-space data, i.e., for each time point the input to the classifier was a vector of amplitudes per sensor. With a sampling frequency of 300 Hz this means that every 3.3 ms a classification accuracy and corresponding vector of regression coefficients were obtained.

Classification accuracy traces after stimulus onset were compared against the accuracies before stimulus onset by cluster-based permutation testing as implemented in FieldTrip (Maris and Oostenveld, 2007). Briefly, this method tests the largest sum of neighboring *t*-values whose corresponding *p*-value exceeded a threshold of 0.05 against the maximum sum obtained when condition labels were reshuffled randomly 1000 times. Because the baseline period was shorter than the post-baseline period, the post-baseline period was divided into three parts and tests against baseline were made for each part separately. The first part was the period between stimulus onset and 500 ms thereafter. The second part spanned from 500 to 1000 ms after stimulus onset. The last part was the period between 700 and 1200 ms after stimulus onset, such that it was of the same length as the previous parts and the baseline. These three separate tests against baseline allowed us to draw conclusions about both classification accuracy during stimulus onset and after stimulus offset, as within a cluster-based permutation test conclusions can only be drawn about the largest cluster per comparison. Multiple comparisons were corrected for with Bonferroni correction. We could not test the baseline period against the chance level of 0.5, as this method does not allow for one-sample tests.

In order to interpret the classification model underlying successful classification, regression coefficients of each classification model were premultiplied with the covariance matrix of the corresponding training data, conform Haufe et al. (2014). This resulted in activation patterns which, unlike the regression coefficients, indicate features that are informative about the identity of the perceived stimulus. As a classification accuracy at chance level indicates that the classifier was unable to discern between the two classes based on the data at hand, the underlying activation patterns cannot be considered to be informative. On the other hand, if the classification accuracy did rise significantly above chance level, the underlying activation patterns can be considered to be informative as well. In this way, the activation patterns derive their significance from the corresponding classification accuracy.

In addition, for each individual subject we trained classifiers on each single time point, and tested them on every other time point to assess the similarity of the data at different time points (King and Dehaene, 2014). This transfer learning was validated by a leave-two-out procedure, leaving out one trial for each class per fold. This ensured that a classifier was never tested on a trial that was also used for training, as within trial similarity may unjustly boost classification accuracy when the same trial is used both in the train and in the test set. Accuracies were tested with a within-subjects *t*-test against the averaged accuracies obtained from transfer learning on time points between 300 and 100 ms before stimulus onset. Multiple comparisons were corrected for using the false discovery rate (FDR).

### Source reconstruction

Sources of the activation patterns were reconstructed with the exact LORETA (eLORETA) solution (Pascual-Marqui, 2007), which has been shown to yield images of current density with exact localization, albeit with low spatial resolution. T1-weighted MRI data were acquired using a 1.5T whole body scanner (Siemens Magnetom Avanto, Siemens, Erlangen, Germany). Vitamin E markers in both ears, in ear molds identical to those containing the head coils during the MEG session, indicated the locations of the corresponding fiducials during the MEG measurement. The location of the fiducial at the nasion was estimated based on the anatomy of the ridge of the nose. These anatomical scans were first resliced to 1 mm slices with a dimension of 256 × 256 voxels, skull-stripped and realigned to the Talairach space. These data were further processed using FreeSurfer's anatomical volumetric processing pipeline and surface-based processing pipeline, which resulted in a reconstruction of the cortical surface. MNE-suite was used to create the source space, i.e., to extract a cortex-restricted mesh of source grid points from this surface. These source locations were then co-registered to sensor space using FieldTrip, by co-registering the volumetric images that were created by FreeSurfer to the sensor array by means of the fiducials. After this, a volume conduction model and leadfield were created. The dipole moment was estimated such that *d* = *Wa*, where *a* represents the sensor-space activations and *W* is the eLORETA filter. This filter was obtained with the previously created leadfield and with the regularization parameter λ of the weighted minimum norm estimation set to 0.05. The power per source grid point was then extracted as the sum of squares of the dipole moment.

For each subject, source grid points were divided into 74 atlas regions of the Destrieux atlas per hemisphere, as extracted with FreeSurfer (Destrieux et al., 2010). The larger atlas areas (i.e., areas larger than 1.5 times the average of the 20 smallest regions) were then further split into smaller regions, as many times as it took to approach the average of the 20 smallest regions. This resulted in 245 atlas regions in the left hemisphere and 250 atlas regions in the right hemisphere, with on average 15.20 grid points included in each region (*SD* = 2.67), rendering the regions more homogeneous in size than before this split (*M* = 54.64, *SD* = 46.14). Activation values per subject for each region were expressed as relative activation increases compared to baseline by dividing them by the averaged activation values obtained between 300 and 100 ms before stimulus onset. We then normalized these values such that for each subject the scale of the patterns was in a similar range, to prevent a skewed influence of some subjects at the expense of other subjects in the group average. We performed this normalization by dividing each relative activation value by the sum of all relative activation values within a subject. These normalized relative activation values were then averaged over subjects.

## Results

### Behavioral results

Reaction times to trials in which letters were probed (*M* = 658 ms, *SD* = 71 ms) were slower than to trials in which faces [*M* = 629 ms, *SD* = 66 ms; *t*_(27)_ = 4.56, *p* = 0.0001] and trials in which objects were probed [*M* = 636 ms, *SD* = 74 ms; *t*_(27)_ = 3.84, *p* = 0.0007]. Reaction times to trials in which faces were probed did not differ significantly from those in which objects were probed [*t*_(27)_ = 1.30, *p* = 0.21]. Likewise, the percentage of correct trials in which letters were probed (*M* = 74.15%, *SD* = 11.65%) was lower than the percentage of correct trials in which faces [*M* = 85.76%, *SD* = 8.49%; *t*_(27)_ = 8.974, *p* < 0.0001] or objects were probed [*M* = 83.27%, *SD* = 10.16%; *t*_(27)_ = 6.55, *p* < 0.0001]. However, when corrected for multiple comparisons (Bonferroni correction with six multiple comparisons), the percentage of correct trials in which faces were probed did not differ significantly from that of trials in which objects were probed [*t*_(27)_ = 2.12, *p* = 0.04 (uncorrected)]. This suggests that, in terms of the memory task, letters were more difficult to remember than both faces and objects, which were equally difficult to memorize. This difference in reaction times and memory performance for letters compared to the other categories is likely due to the letter stimuli being more similar to each other than faces and tools were.

### Classification per time point

First, classification was performed on each individual time point (Figure 1B). Comparing the accuracies to the baseline accuracies obtained before stimulus onset reveals that for the face-letter contrast the significant cluster starts 73 ms after stimulus onset (mean accuracy = 0.53, *SD* = 0.07; summed *t* = 940.31, *p* = 0.001). For the face-object contrast, this onset occurs as early as 63 ms after stimulus onset (mean accuracy = 0.54, *SD* = 0.07; summed *t* = 954.02, *p* = 0.001). Finally, for the letter-object contrast, the onset of significant information is observed at 70 ms after stimulus onset (mean accuracy = 0.51, *SD* = 0.06; summed *t* = 719.44, *p* = 0.001).

Accuracy peaked at 123 ms after stimulus onset for the face-letter (mean accuracy = 0.69, *SD* = 0.13) and 133 ms after stimulus onset for the face-object contrast (mean accuracy = 0.72, *SD* = 0.09). For the letter-object contrast, accuracy peaked after 180 ms (mean accuracy = 0.63, *SD* = 0.06). After this initial peak, accuracies decreased but remained well above chance level throughout the presentation of the stimulus for the face-letter and face-object contrasts. Of note is that for the face-object contrast accuracies decreased more strongly before rising again than was the case for the other contrasts.

When the stimulus disappeared from the screen, neural evidence for the perceived stimulus class did not vanish. For the face-letter contrast, information was still detected for most of the remainder of the analyzed trial, lasting until 620 ms after stimulus offset (mean accuracy = 0.58, *SD* = 0.07; summed *t* = 715.44, *p* = 0.001). Similarly, stimulus information also remained present for 677 ms for the face-object contrast (mean accuracy = 0.55, *SD* = 0.07; summed *t* = 762.67, *p* = 0.001). For the letter-object contrast, information was only present until 277 ms after stimulus offset (mean accuracy = 0.54, *SD* = 0.06; summed *t* = 486.99, *p* = 0.001).

Directly after stimulus offset, the accuracy traces appeared to peak slightly before decreasing. This offset peak occurred 73 ms after stimulus offset for the face-letter contrast (mean accuracy = 0.67, *SD* = 0.09), after 83 ms for the face-object contrast (mean accuracy = 0.66, *SD* = 0.08), and after 90 ms for the letter-object contrast (mean accuracy = 0.64, *SD* = 0.05). Note that, again for the face-object contrast, accuracies dropped directly after this peak before returning upwards again. For the letter-object contrast, the offset peak seemed to be larger than those observed for the face-letter and face-object contrasts. Because of the difference in task-difficulty for the letter-class, however, it cannot be discerned whether this was related to a difference in perception or encoding, or whether this larger peak was due to a task-difficulty effect.

**Figure 2:**
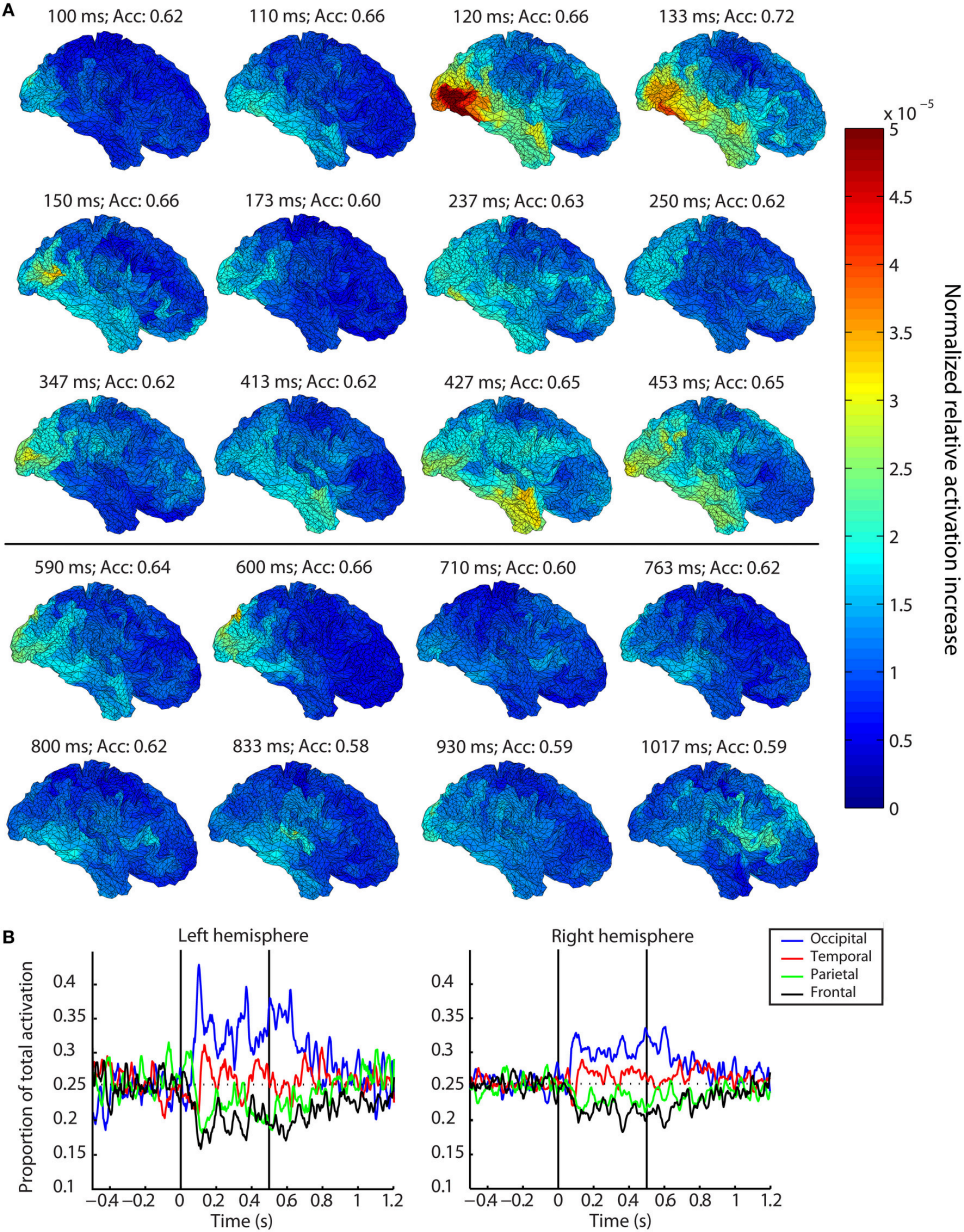
**Spatiotemporal dynamics of visual perception. (A)** Localization of activation patterns at selected time points for the right hemisphere averaged over subjects. Warmer colors indicate a larger average normalized relative increase in activation compared to baseline. Areas with large values contain information about the identity of the perceived or remembered stimulus. Figures above the black horizontal line correspond to time points during stimulus presentation, figures below this line belong to time points after stimulus offset. **(B)** Smoothed traces of the proportion of total averaged normalized relative activation originating from each lobe over time. The first black vertical line indicates stimulus onset, the second line coincides with stimulus offset. The horizontal dashed line indicates 0.25, which indicates an equal distribution of activation for the four lobes.

Classification on the eyetracker and EOG data, from which eye motion components were not cleaned with ICA as was the case with the MEG data, was possible for the face-letter contrast between 240 and 948 ms after stimulus onset (maximum accuracy 0.64 at 450 ms after stimulus onset; summed *t* = 327.85 and 598.98, both *p* = 0.001), for the face-object contrast between 243 and 983 ms after stimulus onset (maximum accuracy 0.62 at 510 ms after stimulus onset; summed *t* = 341.66 and 714.68, both *p* = 0.001) and for the face-object contrast between 780 and 800 ms after stimulus onset (maximum accuracy 0.55 at 183 ms after stimulus onset; summed *t* = 18.23, *p* = 0.01). However, as these data contained eye motion components which have, at least to a certain degree, been removed from the MEG data, it is likely that for the eyetracker and EOG data classification was driven by eye motions that were no longer present in the actual MEG signal. To assess the likelihood that eye motions were driving classification on the MEG signal, we correlated the accuracy traces of the EOG and eyetracker data with the traces obtained when applying the classifier to the MEG data. For none of the contrasts these correlations were significant at any time point when correcting for multiple comparisons with the FDR (face-letter: all *p* > 0.0003, all absolute *r* < 0.63; face-object: all *p* > 0.0028, all absolute *r* < 0.54; letter-object: all *p* > 0.0007, all absolute *r* < 0.60; FDR-corrected alpha = 0.0001). Furthermore, as is shown in the topographic representation discussed below (Figure 2A), none of the underlying sources of this over-time classification seemed to originate from sources related to eye movement. These results indicate that even if some eye motion artifacts remained in the MEG data, these were unlikely to drive classification of the MEG signal.

The remainder of the analyses focused on the face-object contrast only, as no difference in task difficulty was detected for these two types of stimuli, thereby excluding task difficulty as a potential confound.

### Localization of activation patterns

In order to identify what brain areas were involved in the distinction between the perception of faces and objects at each specific time point, and hence which areas could successively be thought to contain information about the stimulus identity, we assessed the model underlying the aforementioned classification accuracies. These spatiotemporal dynamics of perception are shown for a subset of salient time points in Figure 2A and in the Supplementary Movie. Time points during which the group-level accuracy was not significant, and hence during which the underlying activation values were not reliable, are indicated in red.

Specifically, we observed a spreading of activation over time, starting in occipital cortex within 100 ms after stimulus onset. At that time point, mainly occipital areas were activated, such as the occipital pole, superior occipital gyrus, and lingual gyrus. Starting at 103 ms after stimulus onset, activation was found to be spread out more anteriorly, to also include parts of the inferior and superior temporal gyrus, as well as the fusiform gyrus. This activation of both occipital and temporal areas continued with increasing activation of both lobes for the next 17 ms, and then started to decrease again, until 160 ms after stimulus onset. During this period also parts of the frontal lobe were activated, such as the superior frontal gyrus. After that, activations were restricted to the occipital lobe again.

This occipital-temporal pattern repeated itself throughout the remainder of stimulus presentation, albeit with lower and more diffuse activations. This happened for example between 200 and 250 ms after stimulus onset, and between 340 and 500 ms after stimulus onset. During this last peak in activation, the relative weights of the activations in temporal and occipital lobe shifted. Whereas previously activations were strongest in occipital lobe, in this last case the pattern reversed, with little activation in the occipital lobe at 413 ms after stimulus onset. In the temporal lobe, however, activations were stronger and spread mainly toward the most anterior parts of the superior temporal lobe, even including the temporal pole.

After stimulus offset, activation patterns initially mimicked those during stimulus presentation, including mainly occipital and temporal lobe, with most of the weight on the occipital regions. It seemed temporal activations were not as pronounced as during the same time period after stimulus onset. For example, during the peak of accuracies at 83 ms after stimulus offset, the largest activations were observed in the occipital lobe whereas activations in the temporal lobe were less strong. In comparison, the corresponding peak after stimulus onset at 133 ms involved strong activations in both occipital and temporal lobe. This focus on occipital lobe was especially noteworthy for example at 763 ms after stimulus onset (246 ms after stimulus offset), where despite the absence of class-specific stimulus input for more than 200 ms activations were still mainly observed in occipital areas.

In contrast to the post-onset period, starting 183 ms after stimulus offset a distinct frontal component localized around the opercular and triangular part of the inferior frontal gyrus became activated. This frontal component was observed throughout the period where accuracies remained above chance level, occasionally accompanied by activations in occipital and inferior parietal areas. However, it should be noted that this inferior frontal component, albeit specific in time, was driven solely by one subject. Rejecting that subject from the analysis abolished the prominence of this component.

The activation patterns observed for the right hemisphere were very similar to the activation patterns observed for the left hemisphere. However, for the left hemisphere, activation localized to the supramarginal gyrus instead of to the inferior frontal gyrus around 150 ms after stimulus offset. Again, the presence of this supramarginal component was driven solely by a single subject.

The dynamics of the relative importance of the different lobes over time can be seen in Figure 2B. After stimulus onset, activations were predominant in the occipital lobe, followed slightly after this first occipital peak by the temporal lobe. In contrast, activations in the parietal and frontal lobe each made up less than 25% of the total activation. This order remained the same until about 150–200 ms after stimulus offset when these proportions of dominance changed to a more uniform distribution again, with relatively more activation in parietal and frontal areas than before, and relatively less activation in occipital and temporal areas.

### Similarity of stimulus representation before and after stimulus offset

To further investigate the representation of stimulus information after stimulus offset, we assessed the similarity of cortical activity during the onset and the offset peak with transfer learning. In this procedure, a classifier was trained on data during one time point, and tested at every other time point. This can be seen as a test for pattern similarity, where high accuracies indicate that the patterns at a time-point pair are highly similar. The emerging temporal patterns can be informative about the extent to which an underlying process at a given time point is also present at another time point, or whether it is already replaced by a different process. Given the notion that similar processes would underlie transfer learning, a lack of transfer learning over different time windows would suggest different processes playing a role at these different points in time, whereas any distinct block of high transfer learning values would indicate a similar underlying process (King and Dehaene, 2014).

**Figure 3:**
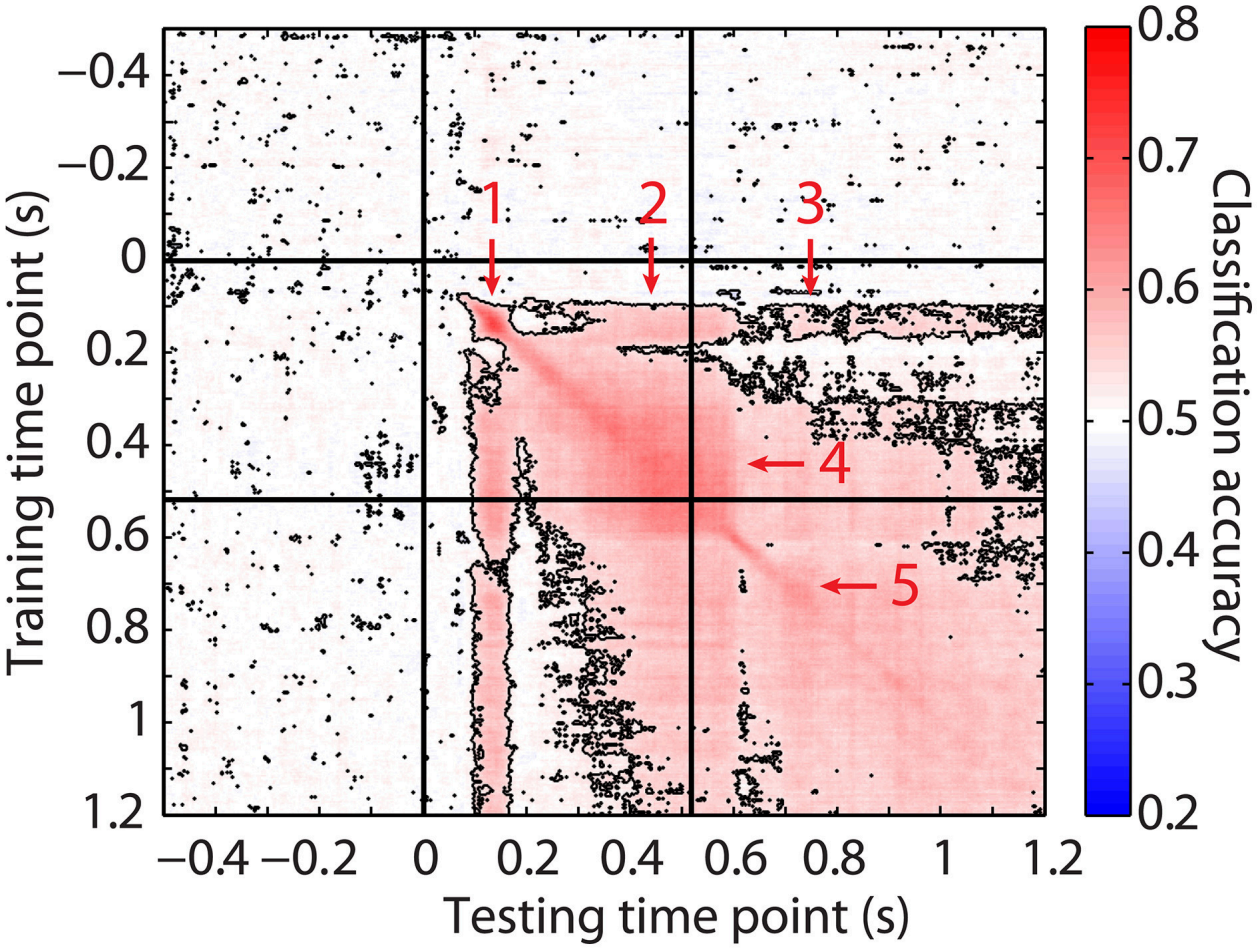
**Transfer learning results**. Average accuracies obtained when classifiers were trained on the MEG signal at the time points on the y-axis, and tested on signals at the time points on the x-axis. Higher accuracies were marked as more red. Outlined accuracies were significantly higher than the averaged accuracies obtained from transfer learning on time points between 300 and 100 ms before stimulus onset (FDR-corrected). The left vertical and upper horizontal line indicate stimulus onset. The right vertical and lower horizontal line indicate stimulus offset. Arrows indicate different blocks of transfer learning that are elaborated on in the text.

As can be seen in Figure 3, transfer learning accuracies rose above baseline for several sets of time-point pairs. First, training and testing on the time points around the onset peak (see Figure 3, arrow 1) resulted in a distinct period of high, above-chance accuracies for transfer learning between 70 and 180 ms after stimulus onset. This coincided with the peak of high accuracies after stimulus onset. The focality of this set suggests that a transient, distinct neuronal mechanism is at work that ends directly after this peak. Interestingly, however, there was significant transfer learning when training on the time points along this initial peak and testing on time points later during stimulus presentation (from 313 ms onwards; arrow 2) and even well after stimulus offset. This transfer learning was strongest until about 610 ms after stimulus onset, before disappearing and reappearing 710 ms after stimulus onset. From this time point onwards, transfer learning remained above chance level, but not as strongly as before (arrow 3). This band of above-chance classification accuracies was not observed when training on the time points directly after the first peak, suggesting that although the neuronal process observed during this peak was observed later during perception and memorization as well, this was not the case for the processes directly following the peak (until about 410 ms after stimulus onset). These processes seemed to be specific to stimulus perception instead.

From about 320 to 590 ms we observed a broad generalization within this time window (arrow 4). This is suggestive of a single process that was going on over this prolonged period of time. Alternatively, this block being much wider than the initial block around the onset peak may suggest that by this time the timing of the underlying neuronal processes had become more temporally jittered, whereas this was still more strictly tied to stimulus onset during the onset peak.

Finally, after stimulus offset there is a final block of largely generalizing transfer learning that started around 650 ms and spanned the remainder of the period after stimulus offset (arrow 5). Again, the large time period showing successful transfer learning could suggest that the same neuronal process was happening during the entire period after stimulus offset. Alternatively, it is not unlikely that the underlying processes were not very tightly time-locked to stimulus onset.

## Discussion

In this study we investigated the spatiotemporal dynamics during and after visual perception. We showed that activation patterns signifying visual category information were first present in the occipital lobe. Subsequently, this spread out toward more anterior regions to include the temporal lobe. After this, activation was solely observed in the occipital lobe again. This pattern occurred for the first time within the first 170 ms after stimulus onset. During the remainder of stimulus presentation this pattern of occipital involvement followed by the inclusion of temporal regions continued. The activation patterns did, however, become more diffuse during the late stage of stimulus presentation. After stimulus offset, category information did not disappear from the brain signal until 677 ms after stimulus offset, although this information did seem to be processed in a different manner from about 200 ms after stimulus offset onwards, as the activation patterns were more equally distributed over all lobes during this last phase.

### Onset of visual category information

A distinction between faces and objects could be made based on the MEG signal as early as 63 ms after stimulus onset, with classification accuracies peaking at 133 ms. The timing of this peak resembles the one observed for the same contrast in a previous study with the same stimuli corrected for spatial frequency (Van de Nieuwenhuijzen et al., 2013). However, the onset of representation-specific information was earlier than in the previously mentioned study, and falls within the lower range of onsets found for a host of other types of visual stimuli and visual perception paradigms. These studies have found the first signs of stimulus-specific information between 70 and 135 ms after stimulus onset (Liu et al., 2009; Simanova et al., 2010; Carlson et al., 2011, 2013; Bode et al., 2012; Van Gerven et al., 2013; Clarke et al., 2015), although not with the temporal precision of 3.3 ms we used in this study.

These results by no means suggest that there is no stimulus information present in the brain within 63 ms after stimulus onset. However, it does mean that a distinction between different classes could not yet be made based on the data. This could be because information is similar for the different stimulus classes during that period, or because the information at that moment still resides in brain areas from which it is not possible to measure properly with MEG. For example, information about visual stimulation is only thought to progress out of the lateral geniculate nucleus of monkeys after about 30–50 ms (Thorpe and Fabre-Thorpe, 2001), rendering it invisible to MEG until that moment.

It should, of course, be noted that the definition of onset and offset of category information is based on the interpretation of significant clusters, which is heavily dependent on the reliability of the accuracy traces, as well as on the way the cluster-based permutation test defines inclusion of a value into a cluster. As such, a seemingly later onset of discrimination between faces and letters (73 ms) compared to the discrimination between faces and objects (63 ms) does not necessarily mean that objects are processed faster than letters. This alleged difference might simply be due to larger inter-subject variability in accuracies, or lower signal-to-noise ratio of the neural activity patterns that are used for classification in the face-letter contrast, resulting in a higher threshold for accuracies to be deemed above chance level. The peaks of the accuracy traces do coincide, suggesting that information content is maximal at the same time for these different contrasts.

This initial peak could be driven by differences in event related fields (ERFs), although both the onset and the peak of information occurred well before the face-selective N170 (Bentin et al., 1996). The P1, however, which is also thought to differ based on stimulus category (Taylor, 2002; Itier and Taylor, 2004) could underlie classification during the first 100 ms. The early onset at 63 ms even coincides with the preceding C1 component of the visual evoked response, thought to have an onset latency as short as 55 ms (Di Russo et al., 2001).

### Spatiotemporal dynamics of visual category perception

After the initial peak in classification accuracy, although accuracies did decrease, the distinction between faces and objects could still be made throughout the entire period the stimulus was presented. For the face-object contrast accuracies even increased after this initial drop after the onset peak. The brain regions underlying successful classification did, however, change as time progressed.

Although the spatial resolution of MEG is not very high due to volume conduction, it is still possible to gain some information about the underlying spatial distribution of the activation patterns by assessing their source space representations. When stimulus information first became discernible, this was localized predominantly in the occipital lobe, specifically occipital pole, superior occipital gyrus and lingual gyrus. The involvement of posterior parts of the occipital lobe before 100 ms after stimulus onset, especially of the occipital pole, is in line with the generators of the C1 visual evoked response (Di Russo et al., 2001). Moreover, the onset of visual stimulus information in V1 as early as 63 ms after stimulus onset falls almost within the range of 40–60 ms for V1 and within the window of 50–70 ms for V2, denoted by Thorpe and Fabre-Thorpe (2001) as being the latencies for visual processing in monkeys.

Soon after this, the activation pattern expanded rapidly to include more anterior areas, such as inferior and superior temporal gyrus and the fusiform gyrus. This spreading starts to occur around 103 ms after stimulus onset, coinciding with the onset of the P1 of the visual evoked response, which has been thought to originate from the fusiform area (Di Russo et al., 2001). In addition, this anterior progression to include the inferior temporal gyrus resembles the progression along the ventral stream (Mishkin et al., 1983), in which a visual image is processed first based on its basic image properties in the occipital lobe, and later based on more global category information in the temporal lobe. This also suggests that categorical information about the stimulus is not only represented distinctly for faces and objects in temporal cortex based on the higher-order category itself, but that this distinction is already present in the representation of the lower-level images properties. Finally, the onset of anterior temporal activation is almost within the window of 80–100 ms during which the spread to the anterior temporal lobe is thought to be occurring in monkeys (Thorpe and Fabre-Thorpe, 2001).

The activation of the temporal lobe, however, is transient, and activation retreats after peaking in intensity to the occipital lobe within 30 ms. This decrease in activation in downstream areas could be interpreted in terms of thorough processing of categorical information to be only transient. After this deep semantic processing has subsided, bottom-up sensory stimulation dominates stimulus processing again.

Over the course of stimulus presentation, the information flow from occipital to temporal areas and back seemed to repeat itself. However, these later activation patterns were more widely distributed than during the first instance. An explanation for this could be that the timing of the electrophysiological signal becomes more jittered over trials and subjects later during stimulus presentation. This inter-trial and inter-subject jitter would result in more variation in the activation patterns, rendering the averaged localizations later in perception less focal than was the case just after stimulus onset. This is in line with the transfer learning results, which became more spread out over time as stimulus presentation progresses, suggestive of a less time-locked signal (King and Dehaene, 2014). After all, with inter-trial temporal jitter, similar signals will be displayed over various time points in different trials, resulting in decodability based on signal similarity over a larger period of time than would be the case if it were time locked. These blocks of transfer learning, which can be interpreted in terms of time periods with a distinct underlying process, can also be viewed in terms of a static representation. A dynamic representation, on the other hand, would be indicated by an absence of above-chance transfer learning accuracies when generalizing over time. Astrand et al. (2015) have shown a mixture of static and dynamic representations for attention and perception in monkeys. In line with this, we also observe clusters of static, similar, representations interleaved with periods of dynamic representations.

Although during stimulus presentation activations were strongest in occipital cortex, this was different toward the end of the presentation period. During the last 100 ms of stimulus presentation, activation was most pronounced in the anterior temporal lobe and even temporal pole, with activations in the occipital lobe decreased in comparison. As stimulus offset always occurred at the same latency after stimulus onset, this could be indicative of a last attempt to thoroughly process the stimulus before it would disappear from view.

### Visual representations after stimulus offset: Committing the visual stimulus to memory

Information about the perceived visual stimulus category was maintained well after the actual presentation of the stimulus had ended. Indeed, classification accuracies remained above chance level until 677 ms after stimulus offset. This does not mean that after this time all stimulus information has disappeared from the brain. In fact, as all trials included in these analyses were trials in which the subsequent memory task was performed correctly, the presented stimulus has to be successfully retained in working memory. However, categorical information is no longer represented in sensor level amplitudes, or alternatively, images of different categories are no longer represented in a differential manner in MEG time-domain data.

At 83 ms after stimulus offset, a peak in accuracy was observed, similar in shape to the accuracy peak after stimulus onset, albeit somewhat lower in amplitude. In fact, the shape of the offset peak mimics that of the onset peak to such an extent that for the face-object contrast, where there is large drop and subsequent increase in accuracies after the onset peak, this same drop and increase was also observed for the offset peak. For the other contrasts where this decrease followed by increase was not observed after the onset peak this was also not observed for the offset peak. Therefore, the offset peak seems to mimic the onset peak. Such an offset peak has been observed in only some of the aforementioned studies (Carlson et al., 2011, 2013; Ramkumar et al., 2013; Clarke et al., 2015), but not in all (Liu et al., 2009; Simanova et al., 2010; Van Gerven et al., 2013). In these studies, the offset peak seems to be observed in MEG data, but not in intracranial and electroencephalography data, suggesting this effect requires a relatively high signal-to-noise ratio to be detected.

As suggested by Carlson et al. (2011), the offset peak can be explained in terms of an effect similar or related to a visual after-image, caused by the sudden change in perceptual input, resulting in an undershoot of neuronal firing. This is in line with the similarity between the onset and the offset peak. Both are in this case a response to the change in visual presentation. It is in this scenario possible to classify on the offset peak, because the same neuronal population that fired during stimulus presentation is showing an undershoot after stimulus offset. If there was category-specific information encoded in these populations, i.e., when decoding was possible during stimulus presentation, this same information can be detected after stimulus offset, as it is the same neuronal population that is showing an undershoot effect.

If the offset peak is solely driven by the mechanistic response of an undershoot in neuronal firing as described above, one would expect only minimal processing of stimulus information. After all, this processing then only occurs by accident, as a side effect of stimulus offset, without the aim to process stimulus information fully as would be required when it would have a behavioral function.

Assessing the underlying activation during the offset peak revealed that the patterns, albeit with a lower amplitude, resembled those during the onset peak, including both the occipital and temporal lobe, with more weight added to the occipital lobe. This similarity in activation patterns is in line with the above-chance transfer learning accuracies from the onset to the offset peak, suggesting that the underlying processes of these two peaks resemble each other. Previous studies applying transfer learning from stimulus onset to stimulus offset, however, have shown accuracies below chance level when training on the onset peak and testing on the offset peak (Carlson et al., 2011, 2013). These patterns underlie an anti-correlation between patterns during the onset and offset peak as a result of an undershoot of the involved sensor level amplitudes (Carlson et al., 2011). One explanation for this difference could be that the previously mentioned studies have no working memory component in the trials included in the analysis, which is the case for the current study. It could therefore well be that for the previous studies only a mechanistic undershoot effect was detected. In this study, however, after stimulus offset the stimulus had to be memorized. The above-chance accuracies could indicate a continued encoding process in the absence of the actual stimulus, to keep the to-be-remembered stimulus available in working memory. This suggests that the offset peak could in some cases simply be the effect of a mechanistic undershoot, for example when it occurs in the absence of a task (Ramkumar et al., 2013). However, it may also, when the circumstances require it, play a role in the process of committing a visual stimulus to working memory.

As in this study activation restricted to the occipital lobe was still observed 246 ms after stimulus offset, it may seem unlikely that this signifies passive processing of the visual stimulus due to neuronal undershoot, as there has been no visual input that contained class information for over 200 ms to respond to. However, this could be interpreted as the visible persistence phase of iconic memory. This phase has been associated with prolonged visual representations in the occipital lobe, and is thought to last about 150–300 ms after stimulus offset, depending on the specific stimulus properties (e.g., Sperling, 1960; Coltheart, 1980; Nikoliæ et al., 2009; Jacob et al., 2013).

After 183 ms after stimulus offset the activation pattern gradually changes, seemingly to include increased activation in the right inferior frontal gyrus and left supramarginal gyrus, which was observed throughout the remainder of the period after stimulus offset. These areas have been implicated in working memory. Specifically the right inferior frontal gyrus has been associated with visuospatial working memory (Baddeley, 2003). This is in line with the strategy often reported by subjects to keep the image in memory by trying to repeatedly visualize it. In addition, the left supramarginal gyrus has been implicated in the phonological working memory (Paulesu et al., 1993; Salmon et al., 1996; Heinrichs-Graham and Wilson, 2015), as well as in retrieval of episodic memories together with the neighboring angular gyrus (Wagner et al., 2005; Vilberg and Rugg, 2008; Hutchinson, 2009). Although the task itself was not a verbal task, subjects did report afterwards to have memorized objects and features by naming them to themselves, which could explain the involvement of a phonological working memory system. However, although this effect fits well within this framework, it should be noted that it is driven by a single subject, and hence should be interpreted with care. This effect does seem to be specifically related to the time period at the very end of each trial, and therefore it is still possible that these frontal components display a genuine, albeit uncommonly observed, process. Further research should assess whether this is the case, or whether this is a spurious finding.

Importantly, activation in the aforementioned areas does not only mean that these regions play a role in working memory. As they underlie successful classification, the identity of the stimulus kept in working memory is represented in these regions. Hence, over the course of memorization of a visual stimulus, the representation first resembles the one also observed during perception of said stimulus, before being committed to another representation that is more distributed over the different lobes. After this, the representation changes yet again, such that it can no longer be detected with MEG and classification is no longer possible.

### Common processes during and after visual stimulus presentation

We observed common processes acting both during and after stimulus presentation. The process underlying the initial peak in information after stimulus onset reoccurred during the late phase of stimulus presentation and even after stimulus offset. Moreover, processes occurring toward the end of stimulus presentation resembled those after stimulus presentation as well. This suggests that stimulus perception and stimulus memorization are not thoroughly different processes, and these common processes in areas related to the previously perceived stimulus may aid in memorization of said stimulus.

However, although there were large commonalities between the processes before and after stimulus offset, they are by no means identical. Especially during the late phase of the delay period activation patterns shifted from those associated with stimulus identification, suggested by the main involvement of occipital and temporal lobe, to memorization, indicated by change to a relatively equal distribution of information over the four lobes. Still, the above-chance level transfer learning accuracies when training on the first 100 ms of stimulus onset and testing on the period well after stimulus offset suggest that in addition to these higher-order processes there is still a continuation of the processes associated with initial stimulus perception.

## Conclusion

We investigated the spatiotemporal dynamics of visual object processing during and after stimulus presentation. Category-specific information was first detected in the occipital lobe within 70 ms after stimulus onset. Information then continued to include the temporal lobe before returning to mainly occipital lobe, after which this pattern repeated itself for the remainder of the period the stimulus was presented. After stimulus offset, cortical representations differed depending on time after stimulus offset. Whereas within the first few hundred milliseconds categorical information was represented comparable to the representation during stimulus presentation, this representation switched to a pattern in which information was more equally distributed over all lobes.

## Author contributions

MvdN, MvG, and OJ designed the study. MvdN collected the data. MvdN and EvdB analyzed the data. MvdN, MvG, and EvdB wrote the paper.

### Conflict of interest statement

The authors declare that the research was conducted in the absence of any commercial or financial relationships that could be construed as a potential conflict of interest.
